# Examining Chronic Inflammation, Immune Metabolism, and T Cell Dysfunction in HIV Infection

**DOI:** 10.3390/v16020219

**Published:** 2024-01-31

**Authors:** Wenli Mu, Vaibhavi Patankar, Scott Kitchen, Anjie Zhen

**Affiliations:** 1 Division of Hematology/Oncology, Department of Medicine, David Geffen School of Medicine at UCLA, Los Angeles, CA 90095, USA; 2 UCLA AIDS Institute and the Eli and Edythe Broad Center of Regenerative Medicine and Stem Cell Research, David Geffen School of Medicine at UCLA, Los Angeles, CA 90095, USA

**Keywords:** HIV infection, chronic inflammation, immune metabolism, T cell dysfunction

## Abstract

Chronic Human Immunodeficiency Virus (HIV) infection remains a significant challenge to global public health. Despite advances in antiretroviral therapy (ART), which has transformed HIV infection from a fatal disease into a manageable chronic condition, a definitive cure remains elusive. One of the key features of HIV infection is chronic immune activation and inflammation, which are strongly associated with, and predictive of, HIV disease progression, even in patients successfully treated with suppressive ART. Chronic inflammation is characterized by persistent inflammation, immune cell metabolic dysregulation, and cellular exhaustion and dysfunction. This review aims to summarize current knowledge of the interplay between chronic inflammation, immune metabolism, and T cell dysfunction in HIV infection, and also discusses the use of humanized mice models to study HIV immune pathogenesis and develop novel therapeutic strategies.

## 1. Introduction

**Figure 1:**
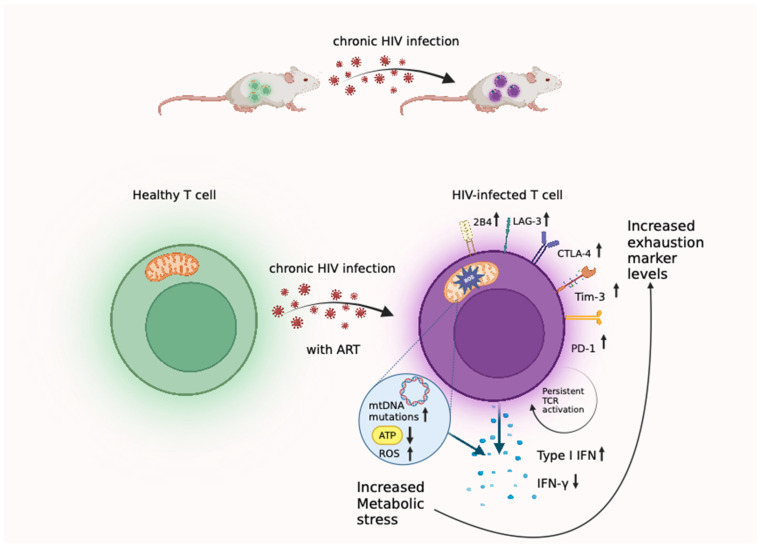
Chronic HIV infection leads to T cells metabolic stress, immune activation and T cell dysfunction. Despite ART, HIV infection induces persistent immune activation and metabolic alterations in T cells, marked by increased Type I IFN, heightened Reactive Oxygen Species (ROS), mitochondrial dysfunction, etc. Both persistent immune activation and metabolic stress eventually contribute to T cell exhaustion.

Chronic human immunodeficiency virus (HIV) infection continues to pose a formidable threat to global health. HIV primarily infects CD4+ T cells, which are crucial for defending the body against various infections and diseases. As the disease progresses in people living with HIV (PLWH), HIV infection is, when compared to uninfected individuals [1,2,3], seen to drive the persistence of higher levels of immune activation and inflammation, which are both a hallmark of the body’s continuous battle against the virus. Despite the success of antiretroviral therapy in controlling viral replication [4,5], there is a growing appreciation that many PLWH, despite successful ART, continue to exhibit signs of chronic, low-grade inflammation [6,7], which is believed to contribute to a range of non-AIDS-related co-morbidities [8,9,10,11,12,13,14]. One of the consequences of chronic inflammation is T cell exhaustion, in which these critical immune cells become less effective, with diminishing capacity to eliminate infected cells effectively [15,16,17]. In addition, the imbalance of metabolic processes, whether directly caused by HIV infection or indirectly by HIV-driven inflammatory responses within immune cells, further contributes to immune activation and dysfunction (summarized in Figure 1) [18,19,20,21]. The combined impact of inflammation, metabolic alterations, and T cell exhaustion underscores the complexities inherent in managing HIV and other HIV-associated disorders [6,22,23]. In this review, we focus on the current understanding of chronic inflammation, immune metabolism, and T cell exhaustion in the wider context of HIV infection in PLWH. We summarize various model system used in HIV research and emphasize the unique advantages of using humanized mice to understand HIV pathogenesis, by using humanized mouse models to investigate the pathogenesis of HIV infection, and especially its relation to immune activation, metabolism, and T cell dysfunction in these novel experimental systems. Understanding these processes is crucial for the development of novel therapeutic strategies that improve the health outcomes of PLWH.

## 2. Chronic Inflammation and Immune Activation Are Hallmarks of HIV Infection

Persistent inflammation in PLWH is characterized by the continuous activation of various immune cells, including T cells [24,25,26,27], B cells [28,29,30], and monocytes [11,31,32,33]; and elevated levels of pro-inflammatory cytokines [34,35,36], including tumor necrosis factor-alpha (TNF-α), interleukin-6 (IL-6), and C-reactive protein (CRP), which contribute to increased morbidity and mortality in PLWH [3,34,37]. Several factors contribute to immune activation/inflammation during chronic HIV infection, including persistent viral replication, microbial translocation, and co-infections with other pathogens [3]. Despite effective, highly suppressive ART, latently infected cells can reactivate and produce new virions, contributing to persistent viral replication [38,39,40]. Certain anatomical locations, such as the lymph nodes [41] and the central nervous system [42], serve as persistent reservoirs, due to limited ART access and ineffective immune surveillance [43], which leads to continuous activation of the immune system and production of inflammatory mediators [44,45]. Additionally, the products of HIV expression induce inflammation by activating various signaling pathways. For example, the HIV protein gp120 has been shown to activate the NF-κB pathway, leading to the production of proinflammatory cytokines [46]. Residual viral particles, as well as Tat and Nef proteins, can also induce cellular activation and the production of inflammatory cytokines [47,48,49,50].

Type I interferons (IFN-Is), including IFN-α and IFN-β, are central components of the innate immune response. IFN-Is are rapidly induced by viral infection through pattern recognition receptors (PRRs) and intracellular proteins that recognize direct cellular infection [51]. HIV infection is rapidly sensed by PRRs and cytosolic sensors that detect viral cDNA or RNAs, which leads to the production of IFN-Is and the expression of IFN-simulated genes (ISGs), which are key effector molecules that exhibit anti-viral activities [52,53,54]. Type I interferon is also critical for the induction of functionally optimal antigen- specific CD8 T cells in HIV infection [55]. However, HIV interferes with the IFN-I responses by impairing functions of ISGs and viral isolates shown to have heightened IFN-I resistance at transmission and ART interruption [56,57]. In addition to their antiviral functions, particularly during acute infections, IFN-Is also have critical immune modulating capacities, and are associated with chronic inflammation in many disease states [51,58,59]. IFN-Is can play a dichotomous role and drive an immunosuppressive and exhausted immune state during chronic infection [59]. Elevated IFN-I-stimulated gene (termed ISG) expression is upregulated in HIV infection, remains elevated despite suppressive ART, and is correlated with disease progression [22,60]. As a result, chronic IFN-I signaling has emerged as a prime suspect in the driving of immune activation and HIV disease progression [59,61,62,63]. Animal study research has shown that blocking type I interferon signaling during chronic infection leads to the restoration of T cell functions and a reduced reservoir [62,64,65]. Additional studies are needed to evaluate if IFN blockade can act as a supplement to ART and improve immune function [66].

Microbial translocation is another major contributor to chronic inflammation [67,68]. Damage to the gut mucosal barrier during acute HIV infection allows the translocation of microbial products, such as lipopolysaccharide (LPS), from the gut lumen into the systemic circulation. This microbial translocation further stimulates the immune system and contributes to systemic inflammation [69]. Lastly, coinfections with other pathogens, such as the hepatitis C virus (HCV), cytomegalovirus (CMV), and mycobacterium tuberculosis, are common in PLWH [70,71,72]. These coinfections activate the immune system, exacerbating chronic inflammation and leading to the increased production of inflammatory cytokines and chemokines, which further drives HIV infection and pathogenesis that can also impact the effectiveness of ART [72].

## 3. Metabolic Stress during HIV Infection

Uncontrolled HIV infection results in progressive CD4 T cell depletion, impairment of both B cell and cytotoxic T cell responses, and ultimately leads to system immune failure and acquired immunodeficiency (AIDS) [73]. Despite the effect of ART, the virus cannot be completely eradicated, and its persistence supports a chronic status of immune activation and immune system dysfunction [22]. As a result, PLWH experience various systemic challenges, including metabolic stress. One of the highly prevalent metabolic dysregulations occurs with lipid metabolism, such as lower levels of high-density lipoprotein (HDL) cholesterol, increased low-density (LDL) lipoprotein, total (TC) cholesterol and triglycerides, leading to dyslipidemia being observed in many PLWH [74,75,76]. Several viral proteins are implicated in dyslipidemia. For example, HIV accessory protein Nef down regulates the adenosine-triphosphate-binding cassette transporter A1 (ABCA1), resulting in reduced efflux of cholesterol to HDL and lipid accumulation in infected macrophages [77]. Moreover, HIV replication is associated with the increase of fatty acid synthase activity, which leads to increased levels of free fatty acids and LDLs [78]. In addition, HIV-mediated immune activation alters lipid processing and transportation, and can lead to production of lipid species that are more ‘inflammatory’, such as oxidized forms of LDL (oxLDL) and HDL (HDLox) [79], forming a vicious cycle of inflammation. Glucose metabolism irregularities, such as insulin resistance [80], which is correlated with coronary artery stenosis [81], are another abnormality associated with HIV infection. Insulin resistance is associated with elevated proinflammatory cytokines and the activation of innate responses, such as toll-like receptors (TLRs), inducible nitric oxide synthase (iNOS), protein kinase R (PKR), c-Jun N-terminal kinase (JNK), and NF-κB, which are connected to insulin receptor and its downstream signaling pathway IRS/PI3k/Akt [82]. Interestingly, a recent study reported that increased monocyte inflammatory responses to oxLDL are associated with insulin resistance in PLWH [83], and noted defects in cholesterol homeostasis and lipid raft impairment are connected to insulin resistance [84,85]. Both findings suggest that factors of metabolic stress are interconnected and exacerbated by systemic inflammation.

Effective ART can, in general, improve the metabolic profile by reducing heightened inflammation and mitigating the inherent effects of HIV replication on metabolism. Nevertheless, patients on ART exhibit significant metabolic stress and antiretroviral drugs can themselves cause metabolic disorders [86]. Classes of ART include nucleoside-analog reverse transcriptase inhibitors (NRTIs), non-nucleoside reverse transcriptase inhibitors (NNRTIs), protease inhibitors (PIs), integrase inhibitors (INIs), fusion inhibitors, and coreceptor antagonists, which each interfere with critical steps in the viral replication lifecycle. NRTIs inhibit DNA polymerase gamma (Pol-γ), which functions in mitochondria DNA (mtDNA) replication and maintenance, and have therefore been implicated in mitochondria toxicity [87]. NRTI may be incorporated into mtDNA via Pol-γ by competing with natural thymidine triphosphate, leading to the mutation or termination of mtDNA. In addition, NRTIs have been shown to impair ATP synthesis, increase oxidative stress, and decrease mitochondria membrane potential Ψm [88] and have, as a result, been linked to long-term metabolic and cardiovascular complications, such as mitochondria toxicity, lactic acidosis, and lipodystrophy [89,90,91,92,93]. NNRTIs inhibit viral replication by binding to a hydrophobic pocket adjacent to the active site of HIV reverse transcriptase. Efavirenz (EFV), a common NNRTI, has been shown to increase oxidative stress, decrease Ψm and induce apoptosis [94]. Protease Inhibitors (PIs), another common class of ART, interfere with the cleavage of essential viral maturation polyprotein precursors by inhibiting HIV protease. PIs, such as ritonavir, have been shown to induce oxidative stress, decrease Ψm and ATP production, and inhibit cholesterol efflux, leading to side effects associated with metabolic disturbances, including dyslipidemia, lipodystrophy, and insulin resistance [89,90,91,93]. Mitochondria toxicity is particularly pronounced in older drugs such as didanosine (ddI) and stavudine (d4T), but is less common in the newer drugs such as lamivudine (3TC), emtricitabine (FTC) and tenofovir (TDF) [95,96]. Nonetheless, 3TC, FTC and TDF were still shown to decrease fat mtDNA content and affect complex I and IV activity levels [97]. The mechanisms of metabolic altercation by HIV infection and ART are complex, multifactorial and not fully understood, and further studies are required to improve clinical management and the healthy lifespan of PLWH.

Metabolic stress driven by HIV infection has a direct impact on immune cell functions [98,99]. Serum and plasma derived from PLWH revealed altered metabolites of lipid and fatty acids, which may play an important role in driving immune dysfunction [100,101,102]. HIV infection-mediated chronic inflammation also leads to increased lipolysis and altered lipid trafficking [103,104], which can lead to the accumulation of lipid droplets in immune cells, and in turn have various effects on their function [105,106]. For example, lipid metabolite long-chain fatty acid inhibits IFN-γ production by stimulating intraepithelial lymphocytes [107], and inhibits T-cell responses by increasing mitochondrial reactive oxygen species (ROS) [100]. Lipid metabolism plays a key role in macrophage function and IFN-I antiviral responses [108,109]. The excessive accumulation of lipid in monocytes can lead to macrophage foam cell formation, which produces high levels of proinflammatory cytokines and promotes atherosclerotic plaque formation [110]. In addition, monocytes and macrophages with excessive lipid also display altered type I IFN responses [111]. Lastly, viral infection and the proinflammatory cytokines TNF and IL-1beta can induce mitochondria stress, resulting in the release of mtDNA and activation of cGAS-MITA/STING, which in turn activates IFN-I and inflammasome signaling [112]. Moreover, damaged mitochondria can also release mtRNA, ROS, and other mitochondria damage-associated molecular patterns (mtDAMPs), triggering innate signaling, leading to further exacerbation of chronic immune activation during HIV infection [113].

Growing evidence indicates that cellular metabolism plays a key role in supporting immune cell maintenance and development, and also guides immune activation and differentiation [114]. Due to these metabolic perturbations observed in PLWH, it is therefore critical to study immune metabolism and its role in HIV pathogenesis and immune exhaustion [115].

## 4. Immune Activation and Metabolic Dysfunction Contribute to T Cell Exhaustion during Chronic HIV Infection

T cell exhaustion is a state of T cell dysfunction characterized by the progressive loss of effector activities, the sustained expression of inhibitory receptors, and metabolic alterations [116]. This phenomenon is observed in cancers and various chronic viral infections, including HIV, and is closely associated with the inability of the immune response to adequately control these conditions [116,117,118]. Exhausted T cells exhibit impaired proliferative ability, cytokine production, and cytotoxic activity, which results in ineffective cellular immune responses [15,116,119]. T-cell exhaustion in chronic viral infections is mainly triggered by the persistent activation of TCR signaling, leading to the increased expression of inhibitory and co-inhibitory receptors, such as PD-1, CTLA-4, TIM-3, 2B4, LAG-3, and CD160 [116,120]. While these molecules have important roles in normal immune functions in acute conditions, their upregulation in chronic conditions, such as HIV infection, are highly associated with immune dysfunction. During chronic HIV infection, the upregulation of inhibitory receptors (or called checkpoint inhibitors) in T cells and engagement with their ligands suppresses T cell activation and function. This persistent inhibitory signaling, combined with altered gene expression patterns, leads to T cell exhaustion and compromised antiviral responses [15,121].

Emerging evidence suggests that metabolic distress also contributes to T cell exhaustion and dysfunction. Healthy immune cells maintain a balanced metabolic state, and primarily rely on oxidative phosphorylation (OXPHOS) in mitochondria for energy production in resting conditions. This metabolic pathway is oxygen-dependent and generates more ATP, compared to glycolysis [114,122,123], and this process supports the basic functions of immune cells without promoting excessive proliferation or activation. When immune cells are activated in response to pathogens or other stimuli, they undergo the “Warburg effect”, a metabolic shift to aerobic glycolysis [124,125]. The intensification of aerobic glycolysis allows cells to rapidly generate energy by converting glucose-derived pyruvate into lactate under normoxic conditions, rather than entering the TCA cycle in mitochondria [126]. This metabolic shift plays a crucial role in supporting biosynthetic demands for the activation and proliferation of T cells [103,127]. Chronic immune activation means that immune cells, including T cells and macrophages, are continuously activated and proliferating, which increases their energy and nutrient demands [124,125]. Interestingly, during acute infection, HIV-1 induces the association of NLRX1 with the mitochondria protein FASKD5 to promote OXPHOS and viral replication in CD4 T cells, and viral load setpoint is positively correlated with the OXPHOS pathway [128]. In contrast, during chronic HIV infection, the metabolic demands can lead to nutrient deprivation and the accumulation of metabolic waste products, which in turn affects T cell functions [21,129]. For example, Loisel-Meyer et al. found that HIV-infected macrophages produce higher levels of lactate, which can accumulate in the tissue microenvironment and contribute to T cell dysfunction [130]. Additionally, ROS production is increased in T cells during HIV infection, contributing to oxidative stress and subsequent T cell dysfunction [131,132]. ART, particularly NRTI-mediated mitochondria toxicity, as described above, can also contribute to decreased mitochondrial OXPHOS activity. Therefore, in chronic HIV infection, OXPHOS is decreased in the peripheral blood monocular cells (PBMCs) of PLWH, and is associated with immune dysregulation [133].

During HIV infection, glucose and glutamine metabolism undergoes significant alteration, in both HIV-infected cells and activated immune cells responding to infection [134,135,136,137]. Compared to uninfected cells, there is increased glucose and glutamine metabolic activity in HIV-infected CD4+ T cells and macrophages [138,139]. Activation of CD4 T cells also leads to increased glucose uptake and the expression of glucose transporters [135,140,141]. Increased glycolytic flux is required for viral production and increases the propensity of CD4 T cells to show apoptosis [130]. As a result, increased glucose metabolic activity and increased Glut1 expression are associated with CD4 T cell activation and depletion during chronic HIV infection in PLWHs, and are not completely normalized by ART [141]. Dysregulation of glutamine metabolism in HIV infection can lead to immune cell dysfunction, as evidenced by a negative correlation between glutamine levels and the production of cytokines and chemokines by CD8+ T cells [142,143]; meanwhile, CD4 cell count is inversely correlated with both glutamine and glucose concentrations [144]. In addition, the metabolism of amino acids such as tryptophan and arginine, which are crucial for immune cell function [145,146], are also impaired during HIV infection. Persistent inflammation and immune activation can lead to the depletion of these amino acids. For example, Indoleamine 2,3-dioxygenase (IDO) is an enzyme that is upregulated during inflammation and degrades tryptophan [147]. Increased IDO activity during HIV infection leads to tryptophan depletion, which can have immunosuppressive effects and contribute to T cell dysfunction [148,149].

Early-stage exhausted T cells exhibit a unique metabolic profile, characterized by reduced glycolysis and increased fatty acid oxidation (FAO); however, these cells exhibited impaired mitochondrial function [21,129]. This metabolic shift from aerobic glycolysis is triggered by continuous antigen exposure, which upregulates the PD-1/PD-L1 pathway, resulting in inhibition of TCR/CD28-mediated PI3K signaling and reduced glycolysis and glutamine utilization in effector T cells [19,150]. In contrast, the terminal stage of exhausted T cells mainly relies on glycolytic metabolism with impaired glycolysis and OXPHOS [151,152,153]. Notably, the decline in glycolysis and the mitochondrial respiration of T cells is observed before the onset of T cell dysfunction in early chronic infection, suggesting that metabolic abnormalities set in before, and not as a result of, T cell exhaustion [19,154]. Therefore, modulating metabolism may provide a feasible and efficient strategy to prevent T cell exhaustion in chronic viral infections.

In summary, immune activation, immune cell metabolic dysfunction and exhaustion are intricately linked in a complex, bidirectional relationship rather than a straightforward cause-and-effect sequence. Immune activation and metabolic dysfunction are direct contributors to T cell exhaustion, while metabolic dysfunction and immune cell dysfunction can further exacerbate immune activation/chronic inflammation. For example, mitochondrial dysfunction can lead to the production of ROS and the release of mitochondrial DNA, which can activate innate immune pathways and contribute to chronic inflammation [113,155]. Understanding the interactions between immune activation, metabolism dysfunction and immune exhaustion during HIV infection is important, and will contribute to research that seeks to generate new therapeutic approaches to HIV infection. Achieving a better understanding of the molecular mechanisms that underlie T cell exhaustion will ultimately help to address current barriers that inhibit the development of more effective therapies. This will however only be achieved by applying various representative models to analyze these interactions.

## 5. Current Approaches to Modeling Pathogenesis and Studying the Antiviral Immunity of HIV Infection

Exploring the interplay between immune exhaustion and metabolic dysfunction in HIV pathogenesis requires a multidisciplinary approach. Various models have a distinct role to play in studying immune activation and metabolic dysfunction that can lead to T cell exhaustion.

In vitro studies that examine primary cells and cell lines exposed to HIV-1 or HIV-1 proteins have provided crucial insights into the mechanisms that drive immune dysfunction [15,156,157], and are essential for understanding the molecular and signaling pathways that are involved in immune activation [15,158,159] and metabolic stress [134,160]. Ex vivo studies, on the other hand, can bridge the gap between in vitro studies and in vivo clinical observations, offering a controlled environment to study the HIV-induced alterations in T cell function and metabolism that contribute to the disease pathology [161,162,163,164,165]. However, both in vitro and ex vivo studies lack the complexity of living organisms and cannot replicate systemic responses. The ultimate source of direct evidence is clinical trials, which provide invaluable data for advancing our understanding of HIV pathogenesis and anti-viral immunity, and grasping clinical implications [166,167], however, clinical research faces practical and ethical challenges, as well as constraints related to the limited availability of tissue sampling.

Alternatively, animal models provide unique opportunities to explore therapeutic and prevention approaches, advance HIV-1 management, and gain insight into the mechanism of HIV-1 pathogenesis. Current animal models for the study of HIV-1 infection include non-human primates and humanized mice. Primate models, owing to a genetic and physiological similarity to humans, are critical for understanding the systemic aspects of immune activation [168,169,170] and viral pathogenesis [171,172,173,174,175]. They have been crucial for observing the progression of T cell dysfunction and realistically depict immune exhaustion in HIV-1 infection [176,177,178,179,180,181,182]. However, the use of nonhuman primate models is constrained by ethical considerations, high maintenance cost, and the limited availability of suitable species, and these constraints have restricted experimental group sizes and limited the assessment of various conditions and parameters. In addition, interactions specific to HIV and human host cells cannot be fully assessed in nonhuman primates, since they are typically infected with simian immunodeficiency virus (SIV) or simian-human immunodeficiency virus (SHIV) [183,184,185,186]. Humanized mice models offer a complementary approach by addressing these limitations and opening up the possibility of investigating interactions specific to HIV-1.

Humanized mice are immunodeficient mice engrafted with human cells and/or tissue that have become increasingly valuable as small animal models, both for the close examination of various human diseases and the development of therapeutic strategies [187,188,189].

When compared to primate models of SIV or SHIV, as well as human clinical trials, humanized mouse models of HIV infection are found to possess all the benefits of small animal models: they raise fewer ethical concerns; are less costly; recapitulate in vivo complexity; permit sampling and intervention that are not feasible in clinical trials; and allow a larger sample size, enabling statistically robust studies, which may not be feasible with primate models. In particular, humanized mouse models reconstituted with human immune cells have achieved significant breakthroughs in improved immune reconstitution and have, in recent years, been increasingly widely used in studies of human immunology, infectious diseases, and tumor therapies [187,188,189]. Humanized mice reconstituted with human T cells and other immune cells can support robust HIV infection and HIV latency; have been widely used to study the biology of HIV infection, pathogenesis and anti-HIV immunity; and have also played a critical role in the testing and development of ART and gene- and cell-based therapeutics [190,191,192,193]. Importantly, the model allows the examination of novel therapies that involve the manipulation of human genetics (such as CCR5 knockdown/gene editing), human cell-based immunotherapy (such as CAR-T cell and NK cell therapy), and human biologics (such as the anti-HIV broad neutralizing antibody (bNAb), checkpoint inhibitor therapy, and cytokine treatment, etc.). As a result, the humanized mouse model has emerged as a popular pre-clinical model. Although the murine drug metabolism is different from the human counterpart, the humanized mouse model still provides a versatile model that can be used to explore immune metabolism, and this is because it more closely approximates to human responses than traditional mouse models.

There are many various types of humanized mouse models, which primarily differ in the background mouse strain and humanization procedure. The humanized mouse models most frequently used for HIV research include:

1. Hu-PBL-SCID mice: This model involves transplanting severe combined immunodeficient (SCID) mice with human periphery blood mononuclear cells (PBMCs) [194]. The hu-PBL-SCID models are susceptible to rapid, potent HIV infection and are therefore good models for studying CD4+ T cell depletion and testing anti-viral compounds [189]. However, the hu-PBL-SCID model’s high susceptibility to developing Graft versus host disease (GVHD) in a relatively short period of time makes it a less than ideal candidate for long-term studies [195].
2. Hu-CD34 mice: hu-CD34 mice are generated by engrafting human CD34+ hematopoietic stem/progenitor cells (HSPCs), isolated from adult bone marrow tissue, adult mobilized peripheral blood, umbilical cord blood, or fetal liver, into immunodeficient mice, such as NOD-*Prkdc*^scid^*Il2rg*^tmiwjl^/Sz (NSG) mice. These mice can support the establishment of a robust human immune system (consisting of T cells, B cells, and myeloid cells, with limited GVHD), and are capable of modeling HIV replication in vivo [189,194]. Although this model supports sustained HIV infection and the establishment of latent/persistently infected cellular reservoirs, the mouse thymus does not support the development of fully functional T cells, resulting in lower levels of T cell reconstitution than the BLT mouse (see below) and non-fully functional T cells. This makes it difficult to study the impact of HIV infection on thymic T cell differentiation and T cell functions [196,197].
3. BLT (humanized bone marrow-liver-thymus) mice: BLT-humanized mice are generated by implanting human fetal liver and thymus tissues into conditioned NSG mice, and simultaneously injecting autologous CD34 HSPCs from a fetal liver [189]. This model allows the development of a robust human immune system, including T cells, B cells, NK cells, and myeloid cells. The humanized BLT mouse model is a powerful small animal model that enables robust human immune reconstitution and robust, natural T cell thymic development, allowing for the comprehensive study of HIV immunity. The model is key to seminal studies of cell and gene therapy and, ultimately, to the discovery of a HIV cure [196,198,199,200,201,202,203,204,205,206,207,208,209,210,211]. It has also contributed to studies of HIV latency [200,212,213,214], and mechanistic studies of HIV immunopathogenesis [64,65,196,215,216,217,218]. Despite its notable advantages, this model presents a number of challenges, including expense, the difficulties of surgical procedures, the procurement of fetal tissues, and the inconsistency between the graft and host disease development [189].

In addition to the aforementioned ways of generating humanized mouse models, the development of new strains of immunodeficient mice has further improved multi-lineage immune reconstitution and the versatility of the humanized mice model [219]. These include but are not limited to, the TKO (C57BL/6 Rag2−/−γc−/−CD47−/−) strain, with deleted CD47 to induce tolerance and reduce GVHD development [220]; MISTRG (C;129S4-*Rag2^tm1.1Flv^ Csf1^tm1(CSF1)Flv^ Csf2*/*Il3^tm1.1(CSF2,IL3)Flv^ Thpo^tm1.1(TPO)Flv^*.

*Il2rg^tm1.1Flv^* Tg(SIRPA), harbors humanized knock-in alleles M-CSF, IL-3/GM-CSF and TPO, and supports improved innate responses and myeloid differentiation [221]; NSG-SGM3 (NOD-scid IL2Rg^null^-3/GM/SF), carries human IL-3, GM-SF and CSF and allows stable myeloid lineage engraftment [222,223,224]; NSG-Tg(hIL34) carries humanized IL-34 and allows the improved engrafting of microglial cells [225]; NSG-Tg (hIL15) carries humanized IL-15 and allows improved Treg and natural killer cell development [226,227]; NSG-A2 expresses human HLA class I A2 molecule supports development of A2 restricted human T cells [228]; and DRAG, which are NOD.Rag1KO.IL2RccKO mice that express HLA-DR4 (0401), shows improved B cell and IgG reconstitution [229].

Each of these models has its own advantages and limitations, and the choice of model depends on the specific research questions being addressed. Among them, hu-CD34 and BLT humanized mice can sustain a chronic HIV infection, which allows researchers to study the long-term interactions between HIV and the human immune system in vivo. Several studies that use humanized mice models have shed light on the mechanisms underlying chronic inflammation and immune exhaustion during HIV infection, with particular emphasis on type I interferon signaling, checkpoint inhibitor expression, inflammasome activation, and cellular metabolic processes. By using the humanized NSG-BLT mouse model, we [65], and others [61,64,230], showed that the chronic immune activation and T cell dysfunction seen in BLT mice after HIV infection resemble the patterns observed in HIV+ patients [231,232]. Importantly, we and others [61,64,65,230] also showed that blocking persistent IFN-I signaling in vivo restored dysfunctional anti-HIV specific T cells, lowered viral loads, and reduced the HIV reservoir. Moreover, our recent study demonstrated that modulating type I IFN signaling with autophagy inducer rapamycin in HIV- infected humanized mice led to decreased immune activation, improved anti-HIV T cell function, produced faster viral suppression during ART, and significantly reduced viral rebound after ART withdrawal [233], further suggesting the pathogenic role of type I interferon during chronic HIV infection.

In addition to chronic type I IFN signaling and T cell exhaustion, HIV-infected humanized mice have also been demonstrated to have elevated soluble inflammatory markers [234,235], increased inflammasome activation [236] and high levels of immune check point inhibitor PD-1 expression in T cells [237], reiterating what has already been seen in PLWH. This has enabled numerous studies that closely examine many different aspects of HIV-induced inflammation in vivo. Studies have shown that blocking PD-1 with an anti-PD-1 antibody in HIV-infected humanized mice led to enhanced T cell responses and reduced viral loads [208,238]. Studies investigating the role of inflammasome showed that a caspase 1 inhibitor can mitigate inflammasome activation and CD4 T cell depletion, and reduce viral load in HIV-infected huCD34 humanized mice [236].

Growing evidence indicates that humanized mice can also be used to study immune metabolism and related therapeutics. For example, induced high cholesterol levels contribute to the proliferation of T cells and T cell–mediated inflammatory diseases in BLT humanized mice [239]. Guo et al. have, in studies using human CD4 T cell-reconstituted mice, investigated the role of OXPHOS in HIV infection. The study demonstrated that metformin treatment inhibits OXPHOS, which targets mitochondrial respiratory chain complex-I, and suppresses HIV-1 replication in both human CD4+ T cells and HIV-infected humanized mice [128,240]. HIV infection also leads to lipid accumulation and increased OXPHOS in HIV-infected macrophages that use humanized mouse model [241]. HIV-infected humanized mice also showed gut barrier dysfunction, and elevated plasma and gut tissue oxidized lipoproteins [234]. Our collaborative studies demonstrated that a treatment (apolipoprotein A-I mimetic synthetic peptides designed to mimic apolipoprotein; and A-1 to remove excess cholesterol) could attenuate macrophage activation, and reduce systemic and gut inflammation in chronically treated HIV in humanized mice [234,235]. With the recent development of the germ-free humanized mice model, additional studies are now seeking to investigate the contribution of resident microbiota to human specific pathogen infection, including HIV [242]. In summary, humanized mouse models have emerged as a versatile animal model that can be used to support mechanistic and preclinical studies of HIV infection and ART-related metabolic stress and T cell dysfunction, including studies of drug treatment, supplement treatment and genetic manipulation.

## 6. Using the Humanized Mice Model to Study the Function and Exhaustion of Engineered CAR T Cell Immunity against HIV

Humanized mouse models provide an ideal platform to evaluate the therapeutic efficacy of engineered immunity and have been widely used to test immunotherapies for HIV and cancer [243,244,245,246]. They include, but are not limited to, bNAbs treatment, checkpoint inhibitor blockade, cytokine treatment, NK cell, and T cell-based therapies that seek to improve anti-HIV immunity and clear infected cells. The humanized BLT mouse model is a particularly good model for the study of T cell-based therapies because it has a human thymus organoid, enabling natural T cell selection and development within the model. The development of chimeric antigen receptor (CAR T) cell therapies, which have emerged as a promising therapy in recent years because of tremendous success as a cancer treatment, was critically influenced by the humanized mice model. Anti-HIV CAR-T cells are genetically engineered T cells that specifically target antigens on the surface of HIV-infected cells [245]. Unlike cytotoxic T lymphocytes (CTLs), CAR T cells do not rely on the endogenous T cell receptor (TCR) for antigen recognition and can bypass some of the limitations of natural CTLs, such as major histocompatibility complex (MHC) restriction and downregulation by HIV; they also directly target conserved regions of the virus, making it harder for the virus to escape [245], and can be engineered to resist HIV infection [245]. The CARs best-suited to HIV are CD4-based CARs, whose antigen recognition domain is the extracellular domain of CD4, which enables the recognition of HIV gp120 on infected cells [247,248,249,250,251,252]. Others have also reported the effective anti-HIV activity of T cells engineered with CAR designs based on broad neutralizing antibodies [203,253,254,255].

We have used the BLT mouse model of HIV infection to evaluate the efficacy of HSPC-derived CAR-T therapy and closely examine engineered antigen-specific T cell responses. We demonstrated that HSPC-based CD4CAR therapy allowed long-term engraftment and development of functional anti-HIV CAR-T cells, which suppressed viral replication [199,256]. We also found that the HSC-derived CAR-T cells persisted for an extended period in both humanized mice and non-human primates (NHPs) (>2 years) [257], indicating the potential for long-term viral control. Studies of humanized mice have allowed the extensive selection and optimization of CAR designs, which has in turn demonstrated the potential for anti-HIV CAR-T cells to contribute to a HIV cure. These critical findings have in turn paved the way for multiple ongoing clinical trials of anti-HIV CAR therapy (ClinicalTrials.gov Identifier: NCT04648046, NCT05077527, NCT03240328).

Interestingly, in both the humanized mouse and NHP models, CAR T cells also develop exhaustion and lose their ability to control the viral replication [256,258]. Humanized mouse models are ideal model to test various strategies to boost the functions of CAR T cells and prevent immune exhaustion. For example, PD-1 checkpoint blockade may enhance the CTL activity of HIV-CAR T cells [259]. Research of cancer immunology has also provided many potential strategies that could be used to improve CAR-T cell function and T cell mediated control [260]. The most widely studied approach is the blocking of inhibitory receptors or the genetic reduction of the expression of inhibitory receptors, with the aim of enhancing CAR-T cell function [261].

Metabolic remodeling is emerging as a promising method to improve the metabolic fitness of T cells and prevent/restore CAR-T cells from exhaustion. The use of 4-1BB costimulatory receptors has been shown to promote mitochondria biogenesis and OXPHOS of T cells [262], and studies, both by us and other researchers, have shown that anti-HIV CAR T cells with 4-1BB costimulatory domain have superior persistence and anti-viral functions [200,256]. New studies of cancer immunotherapy also indicate that manipulating glucose metabolism may result in beneficial metabolic adaptations. For example, glucose-starved T cells upregulate AMPK activity, which enhances mitochondria respiration and fatty acid usage, resulting in these T cells demonstrating better functions and delaying tumor growth [165,263,264,265].

Additionally, the optimization of amino acid nutritional support, enhancement of mitochondrial function, and modulation of both immune and metabolic checkpoints have emerged as novel ways to boost CAR T therapy [263,266,267,268]. For instance, a recent study has shown that the mitochondrial enzyme isocitrate dehydrogenase 2 (IDH2) reduces carboxylate glutamine in CD8 T cells. Inhibiting IDH2 in CAR T cells does not impair proliferation nor affect the effector function of the T cells, but does promote memory T cell formation and enhance antitumor responses [269]. This is especially relevant to HIV CAR-T cell research because of the chronic nature of HIV infection, and the associated importance of long-term immune cell function and persistence in maintaining immune surveillance. Engineering approaches to overcome the exhaustion of CAR T cell therapy will therefore most likely involve a combination of strategies that target immune and metabolic pathways.

## 7. Conclusions

Much of the complex interplay between HIV infection, inflammation, immune cell metabolism, and immune exhaustion remains a mystery to researchers and, it is in this context that humanized mouse models have a particular value, as a powerful and versatile tool that can be used to model HIV pathogenesis and test potential therapeutics. Further research is needed to explore the impact of metabolic remodeling in helping to alleviate chronic inflammation, prevent exhaustion, and improve endogenous and engineered T cells responses. It is however critical to understand the limitation of humanized mouse models, as the choice of mouse strain and method of construction may impact the level of human immune reconstitution, development of cellular and humoral responses, basal metabolic rate, and GVHD. Additional studies are needed to further improve the model so that it better recapitulates human conditions; this will in turn enable the investigation of the multiple factors that impact HIV immune pathogenesis, such as genetics, co-infections, gut microbiota, and immune metabolism.

## Data Availability

No specific data were generated to support reported results.
